# Assessment of the Timely Administration of Birth Dose Vaccines in Northern Nigeria and Associated Factors

**DOI:** 10.5334/aogh.3743

**Published:** 2022-07-26

**Authors:** Rasheedat Mobolaji Ibraheem, Bilkisu Ilah Garba, Rasaki Aliu, Olayinka Rasheed Ibrahim, Afeez Oyesola Bello, Salihu Sheni Mohammed, Mohammed Baba Abdulkadir, Rabiu Hashim, Lawal Magaji Ibrahim, Grace Ahmed

**Affiliations:** 1 Department of Paediatrics & Child Health, University of Ilorin and University of Ilorin Teaching Hospital, PMB 1515, Ilorin, Kwara State, NG; 2 Department of Paediatrics, Usmanu Danfodiyo University Teaching Hospital, Sokoto, NG; 3 Department of Paediatrics, Gombe State University/Federal Teaching Hospital, Gombe, NG; 4 Department of Paediatrics, Federal Medical Centre, Katsina, Katsina State, NG; 5 Department of Paediatrics, Federal Medical Centre, Bida, Niger State, NG; 6 Department of Paediatrics, General hospital, Ilorin, Kwara State, NG; 7 Department of Paediatrics, Ahmad Sani Yariman Bakura Specialist Hospital, Gusau, Zamfara State, NG

**Keywords:** birth dose, routine vaccination, age interval, timeliness, Nigeria

## Abstract

**Background:**

Lack of a timely receipt of vaccines can cause uncertain immune response and under-vaccination. Hence, timely vaccination is crucial to ensure an infant’s early protection.

**Objectives:**

To identify the age of presentation for the birth dose vaccines, vaccine antigens received and factors associated with vaccination presentation by day one in Northern Nigeria.

**Method:**

A descriptive cross-sectional study involving 1 952 mother-infant pairs enrolled from 5 different states in Northern Nigeria. Data was collected using a questionnaire including the socio-demographic, antenatal care (ANC), delivery details, birth dates, vaccination presentation and birth vaccine antigens received. Data analysis was done with the SPSS-21 software.

**Findings:**

The median age of the infants at presentation for birth vaccines was six (interquartile range 2–16) days. A total of 413 (21.2%) infants were brought by the day of birth (day 0) or the next day (Day one), while one-fifth (20.6%) presented after Day 28. The most frequently received antigen was the *Bacille-Calmette-Guerin* by 1 781 infants (91.2%), oral polio vaccine 1 703 (87.2%), and hepatitis B vaccine birth dose the lowest at 75.1% (1 565). The commonest reasons for delayed presentations were an ill baby (24.7%) and an ill mother (21.9%).

Factors associated with presentation within Day one post-birth were hospital delivery (OR–1.67, 95% CI; 1.28–2.19), firstborn (OR–1.40; 95%CI; 1.02–1.93), Christianity (OR–2.14 95% CI; 1.63–2.81), and mother with tertiary education (OR–1.62, 95% CI; 1.05–2.48).

**Conclusion:**

Timely administration of the birth dose vaccines is low in Northern Nigeria. Furthermore, some babies do not get the required vaccines despite presenting for vaccination due to stockout. Strategies for early neonatal vaccination such as vaccination in hospital suites post-delivery and utilizing relatives/fathers to take the baby for vaccination when a mother is indisposed are imperative.

## Introduction

In Nigeria, the National Programme on Immunization (NPI) recommends that newborns receive a single dose of *Bacille-Calmette-Guerin* (BCG), hepatitis B vaccine birth dose (HBV-BD), and oral polio vaccine (OPV°) at birth [1]. Vaccination is a cost-effective tool for reducing the morbidity and mortality associated with these diseases [2]. Unfortunately, vaccination uptake remains low in developing countries with the highest burden of vaccine-preventable diseases.

Nigeria is a highly endemic country for hepatitis B (HepB) infection, with most infections in children acquired prenatally or in early childhood [3]. The reported median prevalence of HepB for children was 11.5% (range 6.0, 17.0) [4]. Also, Nigeria is one of the 30 high tuberculosis (TB) burden countries, ranked sixth globally and second in Africa [5]. The number of TB case notifications for children under 14 years was 9 386 in 2019 [5], while the mortality rate attributable to TB among the under-5 children in Nigeria was estimated at 40–80 deaths/100 000 population [6]. The country was declared polio-free in 2020; however, continuous surveillance and vaccination are vital [7]. These vaccine-preventable diseases are of public health importance; therefore, it is essential to commence prompt vaccination of the infection-naïve neonates.

The timeliness of birth dose vaccination remains a significant problem in developing countries with weak immunization systems [8,9,10]. Lack of a timely receipt of vaccines causes under-vaccination, uncertain immune response, and difficulties in planning and monitoring immunization programmes. All these challenges have implications for disease prevention. In Nigeria, the vaccination uptake in the Northern part is low compared to the Southern regions [11], which poses an additional risk. Thus, this study sought to identify the timing (age) of presentation for the birth dose vaccination, vaccine antigens received and the factors associated with a timely vaccination presentation in Northern Nigeria.

## Methods

**Figure 1:**
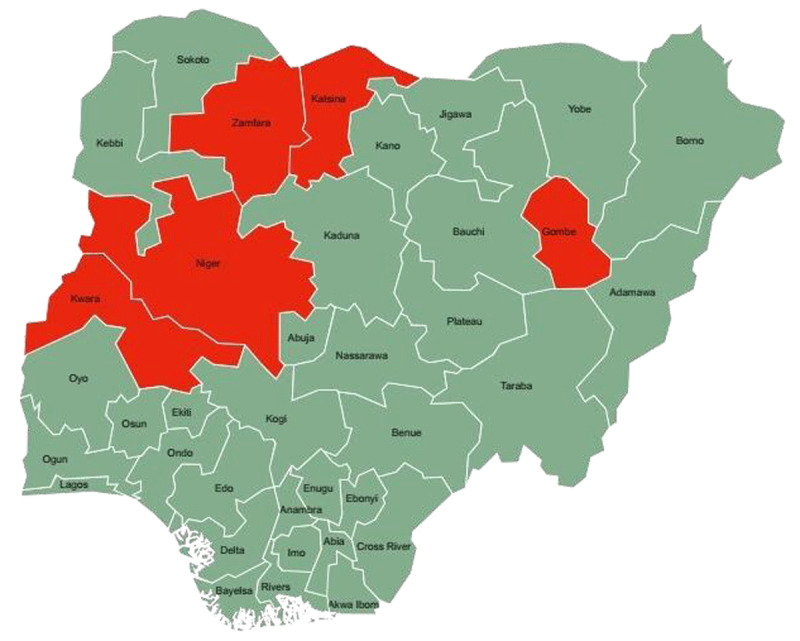
Map of Nigeria shows the study sites’ location (shaded red).

*Study design and setting*: A descriptive cross-sectional study was conducted at 5 immunization centers in five different Northern Nigeria states. The study sites comprised 2 North-Central centers, 2 North-West centers and a North-East center (only 1 center was selected in the North-East because of delays in ethical approval at the second center, and the subsequent COVID-19 lockdown). In the North-West, the study sites were Katsina town in Katsina and Gusau in Zamfara States, respectively. For the North-Central, in Bida, Niger state and Omu-Aran, Kwara State, while in the North-East, Gombe town in Gombe state were selected (Figure 1).

Northern Nigeria consists of 19 states within three geopolitical regions with an estimated population of 104 million based on the 2016 projections of the 2006 national census [12,13]. According to the 2018 Nigeria Demographic Health Survey, the infant mortality rate ranges between 52–104 deaths per 1 000 live births, while the under-5 mortality rate is 74–189 deaths per 1 000 live births [11].

The immunization centers vaccinate infants from Monday to Friday, except on public holidays. For each state, the State Primary Health Care Development Agency (SPHCDA) is responsible for vaccine supply to each local government area. The vaccinators collect the vaccines from a central vaccine store twice to thrice weekly for utilization. The vaccine package consists of multi-dose vials for the OPV (10 doses/vial), Hepatitis B vaccine (10 doses/vial) and BCG (20 doses/vial) vaccines. Also, there are no facility or provider costs to the parent for the vaccines.

*Sample size determination*: This is a secondary analysis of data collected for another study on parental willingness to receive reminders for routine vaccination appointments in North Nigeria; a total of 1 952 mother-infant pairs were enrolled in the study [14].

The eligibility criteria consisted of mothers/caregivers and their newborns at presentation for the birth dose vaccines and with consent to participate in the study.

*Data collection instrument*: This was a semi-structured, interviewer-administered questionnaire deployed in English, Hausa, Nupe or Yoruba languages by research assistants at each center. It had an average time to completion of five minutes. Every mother-baby pair presenting at the immunization center who satisfied the eligibility criteria was consecutively enrolled. The interviewers collected the socio-demographic details of each mother-infant pair, such as gender, mother’s age, marital status, religion, level of education and parental occupation. Responses on ANC attendance, place of delivery of the baby and the birth order of the infant were documented. The date of birth and the date the baby was brought for the birth dose vaccination were recorded. Responses were captured on the reason for the delay in the presentation if the neonate was brought after Day one. Subsequently, the mothers were educated about the importance of vaccination and the number and timing of each vaccination appointment. Enrolment occurred between June 2019 and February 2020.

*Primary outcome measure*- the average age of the infants at presentation for the BD vaccines

*Secondary outcome measures*-

- The proportion of children that received the B.D. vaccines within days 1, 7, 14, 28 and >28
- The proportion of infants that received each of the vaccine antigens
- The reasons for presentation after 24 hours

*Data analysis*: The data was analyzed using the IBM^®^ SPSS version 20.0 (*IBM corporation, Virginia, USA*) 2011. Differences between the date of birth and the presentation day were calculated to identify the interval (in days) to presentation. Timeliness was within Day one (day 0, the day of birth was considered day 0, and Day one, the day after delivery).

The mean (standard deviation, SD) or median (inter-quartile range, IQR) were used to describe the continuous variables. Frequencies with percentages were used to describe categorical variables. The student t-test was used to identify significant differences between continuous variables. A binary logistic regression model was used to identify factors associated with the timely presentation within one day post-delivery. The level of significance was a *p*-value of less than 0.05.

*Ethical Considerations*: The study protocol was reviewed and approved by the relevant Ethical board at each study site (Federal Medical Centre Katsina Ethical Review Committee, Federal Medical Centre Bida Health Research Ethics Committee, Ahmed Sani Yariman Bakura Specialist Hospital Gusau Research and Ethics Review Committee, Kwara State Health Research Ethics Committee and the Federal Medical Centre Gombe Ethics Review Committee). Each caregiver signed the informed consent form after a clear explanation about the study. We did not give incentives to subjects for study participation.

## Results

*Demographic characteristics of mother-infant* pairs—Table 1 shows the demographic features of the mother-infant pairs and the proportion recruited from each site. The mothers’ mean (SD) age was 26.9 (5.4) years. The majority of the mothers (1 848, 94.7%) had ANC, with 1 634 (83.7%) hospital deliveries. The currently married women were 1 923(98.5%), 1 366 (71.0%) in a monogamous marriage and 557(29.0%) in a polygamous marriage. A majority number of mothers (1 569, 80.4%) were Muslims, and 383 (19.6%) were Christians.

**Table 1:** Socio-demographic details of mother-infant pairs.


VARIABLE	FREQUENCY	PERCENTAGE

**Mothers’ age group (years)**		

≤ 20	271	13.9

21–30	1 239	63.5

31–40	417	21.4

>40	25	1.3

**Mothers’ educational level** (n = 1 948)		

No formal education	122	6.3

Primary	83	4.3

Secondary	999	51.2

Tertiary	744	38.1

**Fathers’ educational level** (n = 1 947)		

No formal education	72	3.7

Primary	24	1.2

Secondary	537	27.5

Tertiary	1 314	67.3

**Mother’s occupation** (n = 1 932)		

Professional/large business owner	98	5.0

Nurses/teacher	365	18.7

Artisans	469	24.0

Petty traders	49	2.5

Unemployed/student/housewife	951	48.7

**Mothers’ marital status**		

Single	20	1.0

Married	1 923	98.5

Divorced/Separated	9	0.5

**Birth order of child**		

1st	635	32.5

2nd–4th	988	50.6

≥5th	329	16.9

**Study site**		

Bida	405	20.7

Gombe	384	19.7

Katsina	399	20.4

Omu-Aran	383	19.6

Gusau	381	19.5


The median age of the infants at presentation for the birth dose vaccines was six days (interquartile range 2–16 days). There were 956 (49.0%) males and 996 (51.0%) females.

**Figure 2:**
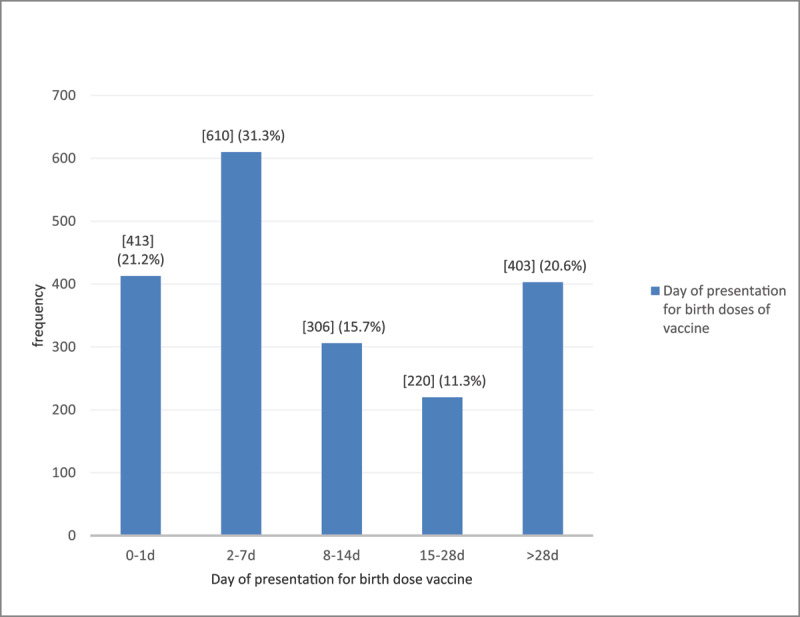
Distribution of babies according to the day of presentation for birth-dose vaccination.

*Time of presentation for the birth-dose vaccination*—The majority number (78.8%) of the infants did not present for birth-dose vaccination within Day one; only 413 (21.2%) infants presented on the day of birth or the next day (Figure 2). One-fifth (403, 20.6%) of the infants were brought for vaccination after Day 28.

**Figure 3:**
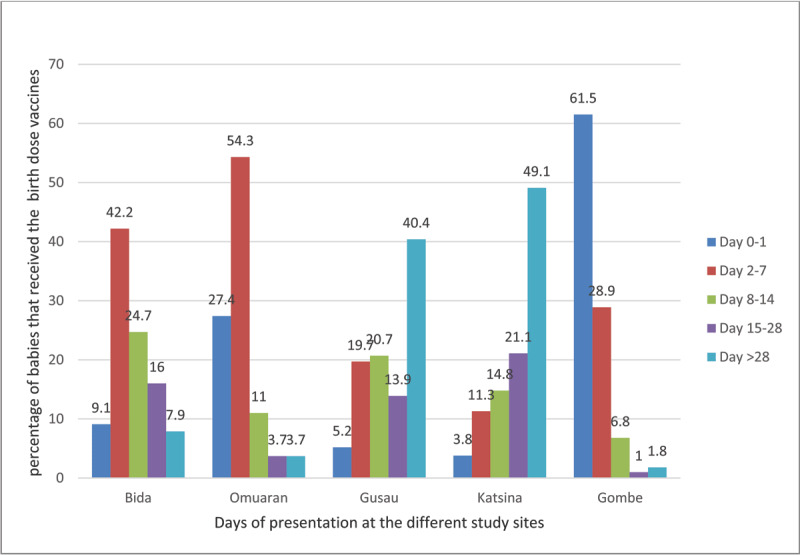
The proportion of babies vaccinated based on the day of presentation at different sites.

Figure 3 shows that Gombe town had the highest proportion of appropriate presentations at 61.5%., followed by Omu-Aran at 27.4%. Katsina and Gusau had the highest proportion of infants presenting after Day 28 at 49.1% and 40.4%, respectively.

Not all the vaccine antigens were available to the 1,952 infants. The BCG vaccine antigen was the most received (91.2%), and the HBV-BD was the lowest at 75.1% (Table 2). Table 2 shows the BCG antigen was the most received at all the 5 sites, with all infants getting the antigen in Omu-Aran, Kwara state and the least (67.7%) obtained in Gusau, Zamfara state. In Bida, Niger state, all the infants received the OPV vaccine, but only 32.1% received this vaccine in Gusau. For each site, more than 80.0% of the infants received the 3 vaccines except in Gusau, where 67.7%, 32.1% and 30.5% of the infants received the BCG, OPV and HBV vaccines, respectively. Also, less than 60.0% of infants received HBV in Katsina. Overall, one-fifth of the children received each birth vaccine within day one of presentation; those from Gombe had the highest proportion for the antigens (61.7%) while Katsina had the lowest (Table 2).

**Table 2:** Proportion of infants that received the vaccine antigens based on study site.


STUDY SITE	BCG VACCINE RECEIVED		OPV° RECEIVED		HBV-BD RECEIVED	
		
	OVERALL N (%)	WITHIN DAY ONE N (%)	OVERALL N (%)	WITHIN DAY ONE N (%)	OVERALL N (%)	WITHIN DAY ONE N (%)

**Gusau (381)**	258 (67.7)	20 (5.2)	122 (32.1)	12 (3.1)	116 (30.5)	12 (3.1)

**Katsina (399)**	365 (91.5)	14 (3.5)	323 (81.0)	12 (3.0)	236 (59.1)	10 (2.5)

**Omu-Aran (383)**	383 (100.0)	105 (26.3)	372 (97.1)	105 (26.3)	359 (93.7)	105 (26.3)

**Bida (405)**	395 (97.5)	37 (9.1)	405 (100.0)	37 (9.1)	370 (91.4)	37 (9.1)

**Gombe (384)**	380 (99.0)	237 (61.7)	381 (99.2)	237 (61.7)	384 (100.0)	237 (61.7)

**Total**	**1 781 (91.2)**	**413 (21.2)**	**1 603 (82.1)**	**403 (20.6)**	**1 465 (75.1)**	**401 (20.5)**


*Reasons for a delay in presentation*—Only 28.5% of mothers proffered reasons for their delayed presentation. Table 3 shows babies’ ill health (24.4%) was the most common reason, followed by ill mother (16.2%) and weekend delivery (12.1%). Unavailability of vaccines was the proffered reason by 6.8% of the mothers.

**Table 3:** Reasons for delay in presentation for birth-dose vaccination.


REASON	FREQUENCY N = 439	PERCENTAGE

**Mother-related**		

Ill mother	71	16.2

Mother had caesarean section	25	5.7

Delayed until after naming	28	6.4

Travelled	17	3.9

Did not know when to come	27	6.1

Forgot	22	5.0

Busy	15	3.4

Lack of transport fare	10	2.3

Father did not want vaccination	5	1.1

Ill husband or sibling	4	0.9

**Access and availability**		

Weekend/public holiday	53	12.1

Vaccine unavailable	30	6.8

Distant vaccination centre	3	0.7

Came on wrong day	10	2.3

**Baby-related**		

Ill baby	107	24.4

Preterm	12	2.7


*Factors associated with vaccination within Day one*: The odds of receiving vaccination were almost 4 times and one-and-a-half times higher among petty traders and teachers, respectively, than the unemployed mothers. Infants of mothers with tertiary education had 1.6 times odds of receiving vaccination within Day one compared to infants of mothers with no formal education. Infants with birth orders less than the fifth had 1.5 times odds of receiving vaccination on day one than those with higher birth order (Table 4).

**Table 4:** Factors associated with presentation within day one.


VARIABLE	B	SE	P-VALUE	95% CI (LL–UL)

**Hospital delivery**				

Yes	0.512	0.138	<0.001	1.669 (1.275–2.185)

No	1			

**Order of birth**				

First	0.340	0.162	0.036	1.404 (1.023–1.928)

Second – Fourth	0.436	0.144	0.002	1.546 (1.167–2.049)

≥Fifth	1			

**Religion**				

Christianity	0.761	0.139	<0.001	2.141 (1.630–2.812)

Islam	1			

**Mothers educational level**				

Primary	0.073	0.308	0.813	1.075 (0.588–1.966)

Secondary	0.292	0.209	0.163	1.339 (0.889–2.016)

Tertiary	0.480	0.218	0.028	1.616 (1.053–2.479)

None	1			

**Occupation of mother**				

Petty Trader	1.373	0.302	<0.001	3.946 (2.183–7.131)

Artisans	–0.096	0.128	0.456	0.909 (0.707–1.168)

Nurses/Teachers	0.427	0.126	0.001	1.533 (1.197–1.965)

Professional	–0.121	0.301	0.688	0.886 (0.491–1.035)

Unemployed	1			

**Maternal age**	0.013	0.011	0.214	1.014 (0.992–1.035)

**ANC attendance**				

Yes	0.106	0.222	0.634	1.112 (0.719–1.719)

No	1			


B = Beta Co-efficient, SE = standard error, CI(LL–UL) = confidence interval (lower limit–upper limit).

The neonates of Christian mothers had twice the odds of neonates with Muslim mothers of getting vaccinated on Day one. Hospital delivery was a determinant of vaccination within Day one, p < 0.001 (Table 4).

## Discussion

The median age of the infants’ presentation for vaccination of 6 days in the index study is higher than earlier reports in North-central Nigeria of one day from Jos [15] and 2 days from Ilorin [16]. The proportion of neonates vaccinated within day one in the current study (21.2%) is lower than the 53.8% and 49.8% reported from Jos [15] and Ilorin [16], respectively. These findings for overall Northern Nigeria compared to the North-central region as highlighted by the earlier studies is a reflection of the poor healthcare-seeking behavior of the North-east and North-west regions of the country [11]. However, it is higher than the 1.3% reported from Benin in South-South Nigeria [9] and 1.1% in the Gambia [10]. These lower findings in the latter studies could be because these sites reportedly had vaccination days either once or twice a week. The need for mothers to wait for that weekday would prevent vaccination within 24 hours postdelivery, thus limiting the proportion of timely newborn immunization as opposed to the current study sites of vaccination on all working weekdays.

The current study’s low timely turnout for birth dose vaccination has significant implications for public health policy and clinical practice. The World Health Organization (WHO) recommends that newborns receive HBV within 24 hours of delivery to reduce the risk of mother-to-child transmission of the virus [17]. This clinical practice recommendation is imperative due to Nigeria’s high disease burden of hepatitis B [4]. Moreover, approximately 90.0% of perinatally-acquired hepatitis B infections progress to chronic disease [18]. Indeed, the timing of the HBV-BD is crucial as the vaccine is 75.0% effective in preventing perinatal hepatitis B transmission when given within 24 hours of birth and 94.0% effective when given with hepatitis B immune globulin [19,20]. Hence, the low uptake of HBV-BD may increase the risk of HBV infection with the likelihood of progression to chronic disease. Concerning TB, the WHO recommends the BCG vaccine at birth or as early as possible to babies in high endemic settings like Nigeria to confer protection against the severe forms of the disease [21]. The delay in BCG vaccination, reported in the index study, increases the risk of exposure to infection and subsequent predisposition to miliary TB and TB meningitis, which have attendant high mortality and neurological sequelae amongst survivors.

A significant reason for the vaccination of neonates after 24 hours in the current study was the mother’s ill health, similar to the report by Sadoh *et al*. [9] and Ibraheem *et al*. [16] Delay in vaccination of the neonates of ill mothers is a missed opportunity, possibly addressed through continuing education of the healthcare providers of sick mothers post-delivery to ensure the infants of these mothers get the appropriate vaccines in a timely way. Other caregivers or relatives can take neonates of sick mothers to receive vaccination with expressed maternal breastmilk if the neonate wants to feed to encourage well-timed birth dose vaccination. Another way to ensure the timeliness of the birth dose vaccine could be via the immediate vaccination of newborns with no acute medical problems. However, this will not take care of home deliveries and deliveries in facilities that do not offer immunization. Illness of the neonate was also a major child-related factor for delays beyond day one in the index study, similar to reports from Ilorin, Nigeria [16] and Vietnam [22].

The most received vaccine was BCG, while the least was HBV-BD in the current study, similar to the report from Senegal of the crude vaccination coverage for BCG, OPV°, and HBV-BD were 95.2%, 89.3% and 88.1% [23], respectively. Vaccine stockout of the birth vaccine antigens was a significant issue in some centres in North-Western Nigeria. This finding is similar to results reported from Jos, North-Central Nigeria [15] and South Africa [24]. The mother who brings her child later than expected for vaccination and cannot vaccinate the child may not return for the vaccination. The implication is thus non-vaccination of the child with increased susceptibility to vaccine-preventable diseases and child death. Indeed, vaccine stockout plays a significant role in causing missed opportunity for vaccination and is a modifiable aspect of immunization. Hence, the need to sustain a pragmatic approach for vaccine purchase, distribution, and availability at the immunization centers. Both vaccinator education in recording number of doses and the use of electronic vaccine stock management to improve stock management have been suggested as means of improving this challenge [24]. These approaches are crucial to enable the country to reduce the burden of viral hepatitis B and sustain the gains in the wild polio eradication certification.

Hospital delivery was associated with a timely presentation for vaccination within day one in the current study, which is similar to findings reported from Benin and Ilorin in Nigeria [9,16] and Senegal [23] but contrasts with the report from the Gambia [10], where hospital delivery was not associated with a timely presentation for vaccination. The contrasting results in the Gambian study may be attributable to the fact that the specific reproductive and child health clinics or outreach clinics give immunizations once or twice a week. The findings of hospital delivery associated with presentation within one day may be due to proximity (babies and their mothers, including the fact that the vaccination points are within the same health care facility) and probably health care workers’ reminders before discharge. Hospital delivery enhances birth dose vaccine administration, especially when seen as part of the routine of postpartum care and has been suggested as one strategy to provide effective vaccine delivery and increase vaccination coverage [25]. Therefore, this finding calls for encouraging hospital delivery, especially in northern Nigeria, where less than half of the women deliver in the health facilities [11]. Furthermore, health education is crucial to the populace to encourage hospital visits for examination and vaccination postdelivery at home. Advocacy for community health workers to carry out home vaccination and postnatal care visits would benefit Northern Nigeria, where most deliveries (50.1–84.4%) occur at home [11]. A strategy utilizing village-based health workers to deliver out-of-cold chain vaccines or prefilled vaccines to infants born at home has reportedly increased the timeliness of the HBV-BD within 24 hours in rural China [26].

Low birth order of the baby had an increased odd of timely birth vaccination receipt, similar to findings from Zaria in Northern Nigeria. However, the latter study used seven days as an indicator of timeliness and reported solely for BCG [27]. Children with a lower birth order were reportedly more likely to consume medical drugs and use inpatient and outpatient medical services [28]. Moreover, mothers of firstborn children were more likely to have better healthcare-seeking behavior due to fear of harming the child, curiosity and eagerness to learn [28] and therefore bring their child for timely vaccinations. Pruckner et al. [28] reported a decrease in vaccination uptake and parental willingness to participate in postnatal health screenings with each additional child after the firstborn. Earlier studies from different countries had also reported timely vaccination of children with low birth order compared with high birth order in the Gambia [29], Iran [30], Saudi Arabia [31], and Greece [32]. The implication for care is to investigate the reasons and address those causes; also, there is the need for consistent education of mothers of children with a high birth order on timely receipt of vaccines to increase vaccination uptake.

In Ethiopia, Boulton et al. [33] reported children from Muslim and Protestant homes had a delayed vaccination for BCG and OPV compared to Orthodox Christians, which is comparable with the current study findings that children from Christian homes were twice as likely as those from Muslim dwellings to have a timely birth vaccination. However, the index study did not differentiate the Christians like the Ethiopian study. Comparing children from Hindu homes (the dominant religion) to Muslim children in India, the latter had 2.2 times greater odds of being non-vaccinated and 1.42 times higher odds of being under-vaccinated than fully vaccinated [34]. This current study finding also contrasts with an observation from Mongolia of children from Buddhist homes being more likely to have timely birth vaccinations than children from homes with no religion, which were the predominant religious inclinations, respectively [35]. The observed differences reported in the index study and the studies above may be due to religious beliefs and practices influencing the reception and uptake of vaccination. For instance, the core Northern states discourage mothers from going out before the child’s naming ceremony, which occurs on the seventh day. Another reason could be that mothers have to seek permission before taking the neonates for vaccination. Delay may occur if the father is not around or refuses to give consent when vaccination is due. The clinical implication for such children with failure to vaccinate immediately after delivery would be a delay in receiving the birth dose vaccines. There is still a pocket of individuals from the Muslim-dominated Northern Nigeria that have the ideology that immunization is targeted at causing harm to Muslims [36]. Hence, advocacy for newborn vaccination at lying-in wards post-delivery or delivery suites are essential. Furthermore, the education of the Islamic religious leaders may play a role in the behavioral change required to ensure timely birth vaccination.

Mothers’ engagement in paid employment had reportedly been identified as a determinant of timely vaccination of infants in Bangladesh [37], which is similar to the above findings of the current study. In contrast, a Northern African study reported the type of employment of mothers did not affect the vaccination rate [38]. Also, a study in China showed that working mothers’ were less likely to present for timely vaccination than non-working mothers [39]. The current findings may be because most working mothers were educated, which aids a better understanding of the significance of immunization. Also, working mothers have an independent income as a source of transport fare. Besides, they are often on maternal leave within the first weeks of delivery, encouraging them to present at the health facility for vaccination. Our findings imply that despite not working, some mothers may not bring their babies for immunization. Hence, the need to sustain health education and campaign on the benefits of vaccinations to all women.

A major strength of the index study is that it was conducted in multiple centres across 3 geopolitical regions in Northern Nigeria. Hence, it was able to identify areas where stockouts were a significant contributor to the non-vaccination of infants.

Limitations include the study sites were located in urban or semi-urban areas that render delivery and immunization services. Hence, it may not wholly represent the rural area. Another limitation is the likelihood of nonresponse bias associated with reasons proffered for presentation after 24 hours, as less than 30% of the mothers responded. Therefore, the finding must be interpreted with caution as the reasons for the delay amongst the nonrespondents are unknown.

## Conclusion

The study identified low timely presentation for birth dose vaccines with variability across Northern Nigeria. Furthermore, some neonates do not get the required vaccines despite keeping vaccination appointments due to vaccine stockouts. Factors associated with delay include ill neonates and mothers. We recommend the need for strategies to focus on early vaccination of newborns, such as vaccination in hospital suites post-delivery, and use of relatives/fathers to take the baby for vaccination when a mother is unable and improved vaccine supply distribution in Northern Nigeria.
